# Who needs ‘lazy’ workers? Inactive workers act as a ‘reserve’ labor force replacing active workers, but inactive workers are not replaced when they are removed

**DOI:** 10.1371/journal.pone.0184074

**Published:** 2017-09-06

**Authors:** Daniel Charbonneau, Takao Sasaki, Anna Dornhaus

**Affiliations:** 1 Graduate Interdisciplinary Program in Entomology & Insect Science, University of Arizona, Biological Sciences West, 1041 East Lowell, Tucson, AZ, United States of America; 2 Department of Zoology, University of Oxford, Oxford, United Kingdom; 3 Department of Ecology and Evolutionary Biology, University of Arizona, Tucson, Arizona, United States of America; University of Sheffield, UNITED KINGDOM

## Abstract

Social insect colonies are highly successful, self-organized complex systems. Surprisingly however, most social insect colonies contain large numbers of highly inactive workers. Although this may seem inefficient, it may be that inactive workers actually contribute to colony function. Indeed, the most commonly proposed explanation for inactive workers is that they form a ‘reserve’ labor force that becomes active when needed, thus helping mitigate the effects of colony workload fluctuations or worker loss. Thus, it may be that inactive workers facilitate colony flexibility and resilience. However, this idea has not been empirically confirmed. Here we test whether colonies of *Temnothorax rugatulus* ants replace highly active (spending large proportions of time on specific tasks) or highly inactive (spending large proportions of time completely immobile) workers when they are experimentally removed. We show that colonies maintained pre-removal activity levels even after active workers were removed, and that previously inactive workers became active subsequent to the removal of active workers. Conversely, when inactive workers were removed, inactivity levels decreased and remained lower post-removal. Thus, colonies seem to have mechanisms for maintaining a certain number of active workers, but not a set number of inactive workers. The rapid replacement (within 1 week) of active workers suggests that the tasks they perform, mainly foraging and brood care, are necessary for colony function on short timescales. Conversely, the lack of replacement of inactive workers even 2 weeks after their removal suggests that any potential functions they have, including being a ‘reserve’, are less important, or auxiliary, and do not need immediate recovery. Thus, inactive workers act as a reserve labor force and may still play a role as food stores for the colony, but a role in facilitating colony-wide communication is unlikely. Our results are consistent with the often cited, but never yet empirically supported hypothesis that inactive workers act as a pool of ‘reserve’ labor that may allow colonies to quickly take advantage of novel resources and to mitigate worker loss.

## Introduction

Complex systems are a broad class of systems in which behavior emerges from the actions and interactions of a number of independent units. These can range from human-made systems such as computer networks [1,2], robot swarms [3,4], transportation networks [5,6], human organizations [7], and economic systems [8], as well as biological systems such as embryogenesis and organogenesis [9], disease transmission networks [10], genes expression networks [11–13], the organization of multicellular systems [14], and social insect colonies [15–17]. In many cases, complex systems are decentralized, self-organized, and optimized for group-level function.

Social insect colonies are highly successful, evolved, self-organized collectives which are often used as models for the organization of complex systems. They are thought to employ sophisticated individual and group-level strategies for the allocation of workers to tasks. For example, individual honey bee workers adjust their foraging strategy according to innate processes, learned information, and social signals [18–21], such that group-level decisions will depend on the amount of stored food [22–24], the rate of food consumption [25], and the food availability in the environment [26]. Thus, task allocation in insect colonies is expected to be flexible to changes in demand for different types of work [27], and robust to individual failure [28,29].

Studies show that experimentally increasing workload, such as by increasing the temperature in honey bee hives [30,31] or increasing food availability [32–34], leads to increased activity and recruitment of new workers to underserved tasks. Additionally, removal studies that simulate worker loss (by death or otherwise) show that the lost workforce tends to be replaced by other workers in the colony [30,35–40]. It is worth noting that, although social insect colonies are generally thought to be highly flexible and robust, there is also evidence that this is not always the case and that rapid worker reallocation does not always occur when conditions change (either by fluctuating workload [41,42] or by worker loss [43,44]).

Despite these sophisticated mechanisms of task allocation, perhaps one of the most surprising features of social insects is that high levels of inactivity are common in most species. Social insect colonies typically have upwards of 50% of their workers inactive at any one time (honey bees [45,26,46], bumble bees [47], wasps [48], termites [49], and ants [50–54]). Although individual activity level may vary over the course of the day, or across days, the relative activity ranks of individual workers are consistent among workers over moderate timescales (one to a few weeks [35,35,50,55–57]), indicating that workers are consistently more or less active.

Although the abundance of highly inactive workers may seem counterintuitive given the sophistication and flexibility of social insect task allocation strategies, it may be that inactive workers actually contribute to colony function rather than impair it. Indeed, the most commonly cited explanation for inactivity proposes that inactive workers are ‘reserves’ that serve as an auxiliary labor force that can help the colony react quickly to workload increases (other proposed hypotheses reviewed in [17,58]). Thus, if inactive workers constitute a pool of workers that can be dynamically allocated to tasks and therefore adjust the amount of work performed by the colony to fluctuating demands, their presence may allow colonies to be increasingly flexible and resilient (see [17] for an extended discussion of this hypothesis).

Studies that experimentally remove workers show that colonies tend to replace lost workers. However, these studies typically only remove key workers such as bees that increase air movement in colonies by fanning [59], workers active in emigration tasks such as scouting new nest locations or recruiting new workers to potential nest locations [57], workers that remove ant corpses from the nest (undertakers) [60], nurses that care for brood [61], and foragers [62,63]. In these cases where workers are identified in only one task, the individuals removed tend to be highly active workers. In most cases cited above, removed active workers are effectively replaced: their tasks and workloads are taken over by other workers and performed at comparable activity levels (though see [43,44]). Replacement can be immediate (e.g. water foragers [62]), but there is typically a delay ranging from a few hours to a few days [57,60–63].

In studies where pre-removal behavior was known, replacement workers tended to be less active, but not inactive, workers previously performing the replacement task at a lower level, who increased their activity levels to compensate for the loss of highly active workers [61,62,64]. Thus, although the ‘reserve’ worker hypothesis is commonly proposed, this is not direct evidence for it, since actually ‘inactive’ workers were not involved. The few studies that claim to support the ‘reserve worker’ hypothesis [35,65] define inactivity as the absence of observation of a set of pre-determined behaviors. Consequently, workers defined as ‘inactives’ may in fact have been performing a wide range of tasks (e.g. patrolling, grooming, and building) that were not part of the pre-determined task list, and so may not have been truly inactive at all. In fact, the only study that has successfully ‘activated’ inactive workers removed all other workers except for the inactives [66]. There is theoretical evidence that such workers could ensure colony survival in the case of a major catastrophe that eliminates or fatigues all other workers [67].

Inactive workers represent a significant investment of colony resources, typically making up more than half of all workers. This suggests the possibility that they play an important yet unknown role within the colony, in which case we should expect them to be replaced if lost. Many potential functions other than as ‘reserves’ have been proposed to explain the presence of highly inactive workers (reviewed in [17] and [58]). These include inactivity as a form of social ‘cheating’ in which egg-laying workers selfishly invest in their own reproduction rather than contribute to colony fitness by avoiding risky tasks and conserving energy by remaining inactive (tested and supported [68,69]; tested, but not supported [70,52,66]), and inactive workers performing an as-yet unidentified function, such as playing a role in communication (proposed in [71]; tested, but not supported [58]) and acting as food stores, or repletes (tested and supported [58,72]). Thus, if inactive workers have a function other than as reserves, we expect the colony to have mechanisms to ensure that workers are allocated to that function just as we see for active workers.

In this paper, we test whether social insect colonies have mechanisms that maintain colony activity levels homeostatically (in the broad sense of returning to a desired value after perturbation) by removing either the 20% most active, the 20% most inactive, or the same proportion of random workers, then tracking the activity levels of the remaining workers post-removal.

## Methods

We collected 20 colonies of *Temnothorax rugatulus* ants (1307 workers total, colony size mean = 65.35, median = 56, s.d. = 33.01) from a pine forest located at ~8000ft in altitude in the Santa Catalina Mountains, USA (N32.395 W110.688) in June and July of 2015 (15 colonies) and 2016 (5 colonies; see S1 Table). The colonies were collected in a National Forest (Saguaro National Park East) which does not require a permit for collections of this scale. The collection consisted of 20 colonies of *Temnothorax rugatulus* ants, an abundant and widely distributed species that is neither endangered nor protected.

Within 1–2 days of their collection, colonies were allowed to emigrate to artificial nests consisting of a piece of cardboard with an enclosed nest area and entrance die-cut out of the middle, sandwiched between two glass slides [50]. Colonies were kept in the lab on a 12h/12h light regime (8am to 8pm) and fed water, diluted honey solution, and wingless fruit flies *ad libitum* (similar methods to [50]).

### Overview of methods

To test the effect of ‘active’ and ‘inactive’ worker removals, we applied one of three treatments to colonies: removal of the 20% most active workers (5 colonies), removal of the 20% most inactive workers (9 colonies), and removal of 20% randomly selected workers (6 colonies; see below for detailed descriptions of worker activity and inactivity measurements). From this point on, we will refer to the 20% most active and 20% most inactive as ‘active’ and ‘inactive’ (in quotations). Whole colonies were filmed over 3 consecutive days before removals (shown to be sufficient to obtain consistent individual behavior [50]), at one week post-removals, and at two weeks post-removal (total of 9 videos per colony). Typical timing of events was as follows: Day 1: colonies collected and re-housed, Day 4: workers painted, Day 6–8: pre-removal videos, Day 9: workers removed, Day 13–15: 1 week post-removal videos, Day 21–23: 2 weeks post-removal videos. There were occasionally delays of up to 1 week between collection and painting, but the schedule was consistently maintained once filming had begun.

### Filming

Each video was 5 minutes long. Colonies were filmed using an HD camera (Nikon D7000 with Nikon AF-S Micro-NIKKOR 60mm f/2.8G ED Lens and Lumix DMC-GH3 with Olympus OL6028 60mm f/2.8 Macro Lens) mounted directly over the nest with three sources of diffused light to reduce shadows. The entire nest, as well as the available food and water outside of the nest, were within the field of view. All videos were taken within ~1 month of colony collection to limit potential laboratory effects, such as artificial age structures due to increased forager age. Previous work has shown that colony time spent on any specific task (including inactivity) do not significantly vary between field and laboratory within these timeframes [73].

### Measuring individual behavior

Within 1 week of colony emigration to artificial nests, each worker was painted with a unique color combination to allow individual tracking between observations (see [50] for detailed methods). Worker mortality from paint marking (typically resulting from painting over antennae, leg joints, or spiracles) was estimated to be fairly low (<5%), but was not systematically tracked. Colonies were filmed at least 48h after workers had been painted to allow colonies to return to their normal function.

Videos were analyzed by tracking each individual worker over the course of the 5 minute video and recording the task the worker was performing for each second of the video (a list of possible tasks can be found in Table 1). Tasks were classified as either ‘active’ (building, foraging, brood care, self- or allo-grooming, trophallaxis, and feeding on dead fruit flies that had been brought back to the nest), ‘undifferentiated’ (walking inside the nest and not otherwise engaged in any active task), or ‘inactive’ (immobile and not otherwise engaged in any active task).

**Table 1:** List of possible behaviors observed during video analysis, their broad class of activity, codes, and detailed descriptions. For every second of analyzed video, each ant has one of these behaviors attributed to it. *[Similar to 73]*.

Class	Task	Definition
**Active**	Foraging	All extra-nest activities: ***Building***—Manipulating a stone in any way (moving, pushing, pulling) OR ***Foraging***—Located in feeding area or on water tube or wandering outside of the nest and not engaged in building. Also if returning to the colony from foraging areas and performing trophallaxis or returning with Drosophila flies (food).
	Brood care	Manipulating brood (feeding, grooming, moving)
	Grooming	Grooming itself OR Grooming another ant OR Be groomed by another ant
	Trophallaxis	Receive or give liquid food to/from another adult ant
	Eating	Feeding on drosophila inside nest (brought back by foragers)
**Undifferentiated**	Wandering inside nest	Anytime an ant is mobile inside the nest wall and not engaged in any ‘active’ task
**Inactive**	Inactive	Immobile and not engaged in any ‘active’ task

Worker activity and inactivity levels were calculated by averaging the proportion of observed time spent on active tasks or inactive, respectively, for videos from each block of 3 consecutive days of filming (pre-removal, 1 week post-removal, and 2 weeks post-removal). Inactivity as defined here is *not* simply the inverse of activity (i.e. the absence of activity) because worker time is distributed among time spent active, inactive, and wandering inside (i.e. undifferentiated activity, which is not counted towards either activity or inactivity). Workers that were observed only once during each block (appeared in only one of the three videos) were removed from the analyses to ensure an adequate representation of worker time budgets. Of the workers with sufficient data, the 20% with the highest activity and inactivity levels were removed for the ‘active’ and ‘inactive’ removals respectively. We selected removals of 20% of workers as this should be sufficient to cause a significant effect on colony activity/inactivity, while ensuring a range of activity/inactivity levels in the remaining workers. Random removals were selected using the R function ‘sample()’.

### Determining worker task groups

Previous work has shown that workers of *Temnothorax rugatulus* ants can be grouped into four distinct task groups, or behavioral castes: inactives, foragers, nurses, and walkers [50,58]. These groups specialize (i.e. spend more time relative to other workers) on inactivity, foraging and building, brood care, and wandering inside respectively (Table 1). Here, we use these tasks groups to determine the role of workers within colonies prior to removals.

Using the mean proportion of time spent on tasks pre-removal, we established pre-removal worker task groups using a combination of principal component and hierarchical cluster analyses (prcomp and hclust, base ‘stats’ package in R Version 3.1.2) as follows. We determined the number of distinct task groups (4) by identifying tasks that contributed the most to the principal components (tasks with absolute sum > 1; number of components determined via parallel analysis [74]. We then used a hierarchical clustering analysis to classify workers into distinct task groups based on similarities in their time spent on tasks (worker clustering is identical to [58]; see S1 Fig). Workers were clustered into 4 distinct task groups: (1) Inactives (605 workers– 40.8%), (2) Foragers (145 workers– 9.8%), (3) Nurses (249 workers– 16.8%), and (4) Walkers (483 workers– 32.6%) (task group names capitalized from this point on). The Inactive task group, obtained via hierarchical clustering analysis, should not to be confused with the ‘inactive’ group, which represents the 20% most inactive workers as determined by the continuous variable of % time spent inactive. In order to account for inter-colony variation in inactivity levels, the data for each colony were centered (task mean subtracted from task values) and scaled (task values divided by task standard deviation) separately before being pooled and clustered. To avoid the effects of sporadic (low repeatability) tasks (such as grooming and eating), we performed the clustering analysis only on highly consistent (high repeatability) tasks, namely inactivity, wandering inside, foraging, brood care. The same groups (Foragers, Nurses, Walkers (then ‘patrollers’), and Inactives were obtained previously via clustering analysis that included each individual task [50]. Removing less repeatable tasks from the analysis allows for clustering that is less dependent on small random variation in tasks that are not representative of worker specialization. These methods have been used to describe colony organization in prior studies [75,76], including on *Temnothorax rugatulus* [50,58].

### Statistical analyses

Statistical analyses were performed in R (Version 3.0.3), and consisted of Mixed-effects models and Tukey post-hoc tests (packages ‘nlme’ v3.1–115 and ‘multcomp’ v1.3–2), as well as Wilcoxon Signed-Rank tests (base ‘stats’ package, ‘wilcox.test’ function). Model formulae can be found in the figure legends, and generally include the predicted fixed effect (typically mean individual worker activity/inactivity–i.e. average amount of time spent active/inactive by an individual relative to total observation time) as well as colony identity as a random effect. The fixed effect ‘Trial’ refers to time point in the experiment (pre, 1 week post, 2 weeks post), while treatment refers to the removal treatment (active, inactive, random).

## Results

The across-colony mean proportion of time workers spent on active tasks was 0.170 (median = 0.154, s.d. = 0.071). The mean proportion of time spent inactive was 0.607 (median = 0.628, s.d. = 0.146) (see S2 Fig for distribution of colony activity levels) which is consistent with previous work on *Temnothorax rugatulus* [50,58,73].

To ensure that worker removals should have a significant impact on colony activity and inactivity levels, we compared the activity and inactivity levels of whole colonies (i.e. mean activity levels of all workers in a colony) pre-removal to the calculated mean colony activity level of colonies without the workers that would later be removed (i.e. mean activity levels of all workers in a colony minus the removed workers). This calculation showed that removal of ‘active’ workers should significantly decrease colony activity level (Wilcoxon Signed-Rank Test, p = 0.031), removal of ‘inactive’ workers should decrease mean inactivity level (Wilcoxon Signed-Rank Test, p = 0.031), and removal of random workers should not affect either colony activity or inactivity (Wilcoxon Signed-Rank Test, p = 0.50 and p = 1.00 respectively; S2 Table). Furthermore, mean colony activity (the average of all worker activity levels for that colony) was less than the mean activity level of the removed ‘active’ workers (Colony = 0.18, Removed ‘active’ workers = 0.42), and mean colony inactivity was less than the mean inactivity level of the removed ‘inactive’ workers (Colony = 0.64; Removed ‘inactive’ workers = 0.75). Workers selected for the random removals treatment had comparable activity and inactivity levels to colony activity levels (Activity: Colony = 0.17; Removed random workers = 0.20; Inactivity: Colony = 0.63; Removed random workers = 0.57; S2 Table).

### Are activity and inactivity levels maintained post-removal?

**Fig 1:**
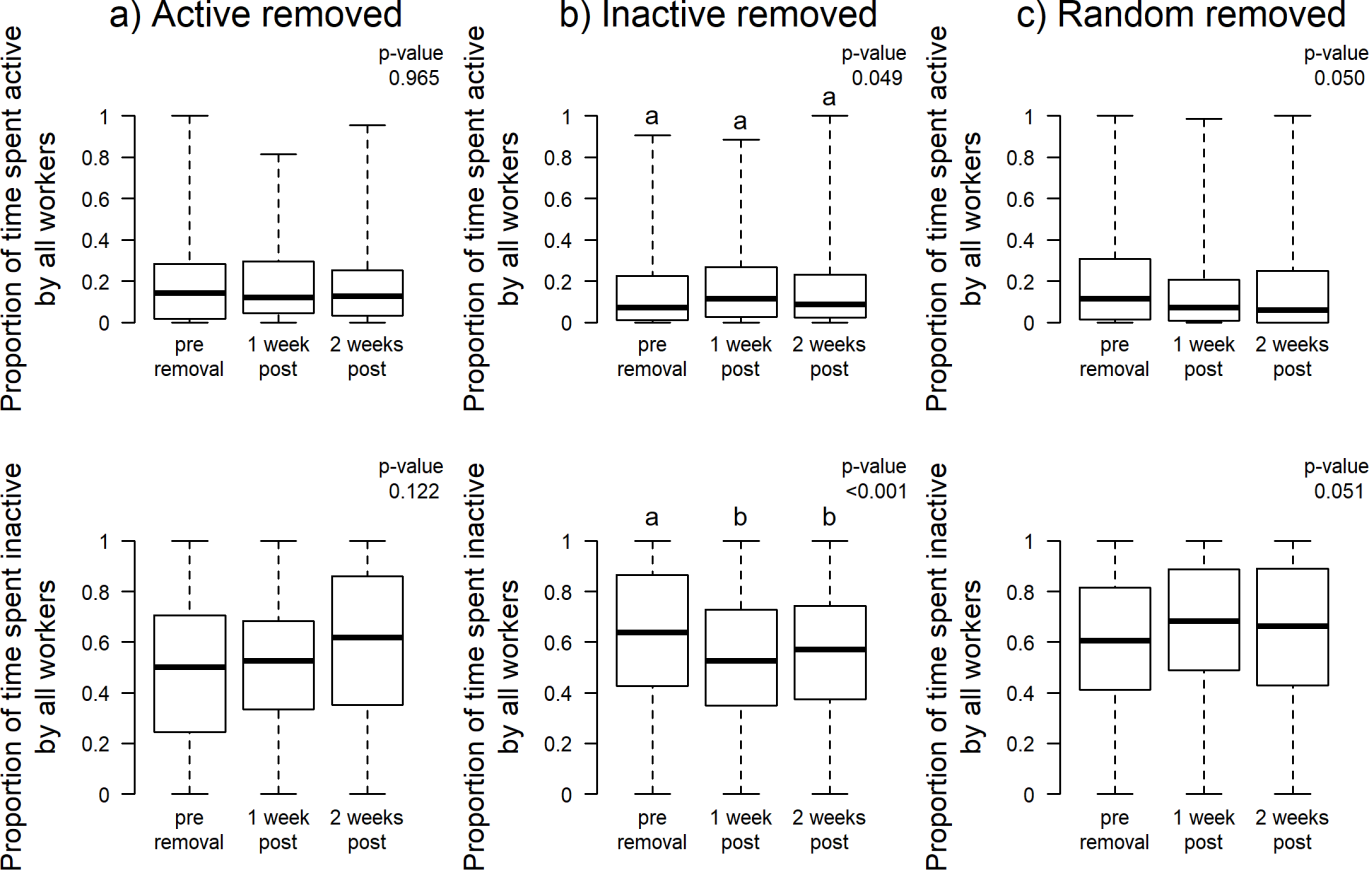
Mean worker time spent active and inactive pre-removal, 1 week after removal, and 2 weeks after removal of (a) active workers (20% most inactive in each colony) (b) inactive workers (20% most inactive in each colony), and (c) randomly selected workers (20% random workers from each colony). Boxplots show median (bar), quartiles (box), and extremes (whiskers) for best illustration (all figures). *Model: *LMM*, *fixed*: *Worker inactivity/activity ~ Trial*, *random*: *Colony–Contrast significance determined using Tukey post hoc tests*.

When ‘active’ workers were removed, neither average worker activity nor inactivity were significantly different from pre-removal activity/inactivity levels (Fig 1A). This is consistent with the idea that the colony rapidly compensates for the removal of active workers.

In colonies where ‘inactive’ workers were removed, there was a non-significant increase in mean worker activity after 1 week and a return to pre-removal activity levels after 2 weeks (p = 0.049, Tukey posthoc shows no significant contrasts). Inactivity levels decreased 1 week after removals and remained low at 2 weeks post-removal (Fig 1B).

There were no significant changes in worker activity or inactivity post-removal of random workers (Fig 1C).

### Are the activity/inactivity levels of the most active/inactive workers post-removal comparable to those of the most active/inactive workers pre-removal?

**Fig 2:**
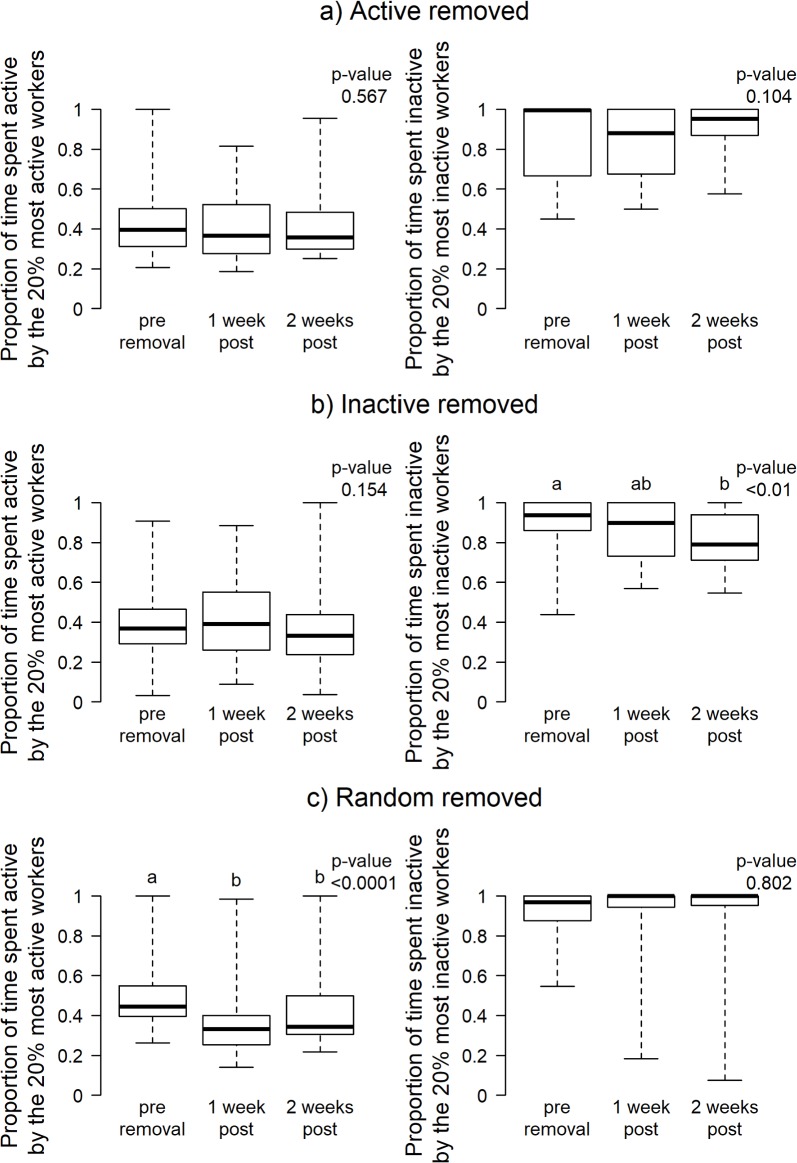
a) (left) Mean time spent active by the most active workers (top 20th percentile) pre-removal, 1 week after removal, and 2 weeks after removal of active workers. (b) (right) Mean time spent inactive by the most inactive workers (top 20^th^ percentile) pre-removal, 1 week after removal, and 2 weeks after removal of inactive workers. c) (left) Mean time spent active by the most active and (right) the most inactive workers (top 20^th^ percentile) pre-removal, 1 week after removal, and 2 weeks after removal of randomly selected workers. *Model: LMM, fixed: Worker inactivity/activity ~ Trial, random: Colony–Contrast significance determined using Tukey post hoc tests.

**Fig 3:**
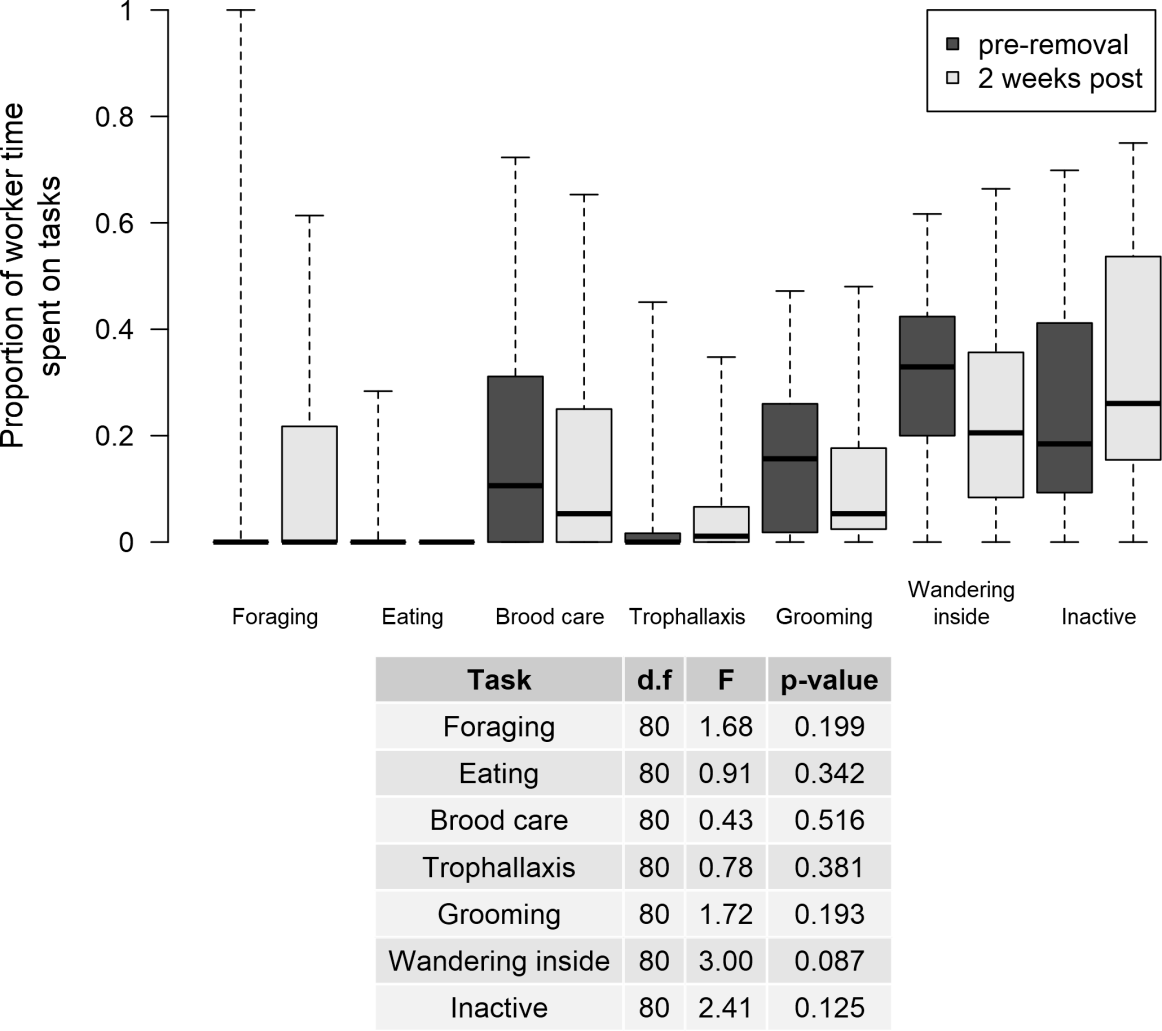
Mean time spent on specific tasks by the most active workers (top 20^th^ percentile) pre-removal and by the most active workers (of those remaining) 2 weeks after removal of active workers. *Model: LMM, fixed: Worker time on task ~ Trial, random: Colony.

In colonies where ‘active’ workers were removed, the activity levels of the most active workers post-removal (top 20^th^ percentile 1 and 2 weeks after) were not significantly different from the activity levels of the most active workers pre-removal (Fig 2A—left). Furthermore, average time spent on specific tasks did not differ significantly between pre-removal and 2 weeks post removal (Fig 3). When ‘inactive’ workers were removed, the most inactive workers post-removal (top 20^th^ percentile 1 and 2 weeks after) were significantly less inactive 2 weeks post-removal than the most inactive workers pre-removal (Fig 2B—right). When random workers were removed, activity levels of the most active workers significantly decreased and remained lowered, while inactivity levels of the most inactive workers were not significantly affected (Fig 2C).

### What role did the most active and inactive workers post-removal hold prior to removals?

**Fig 4:**
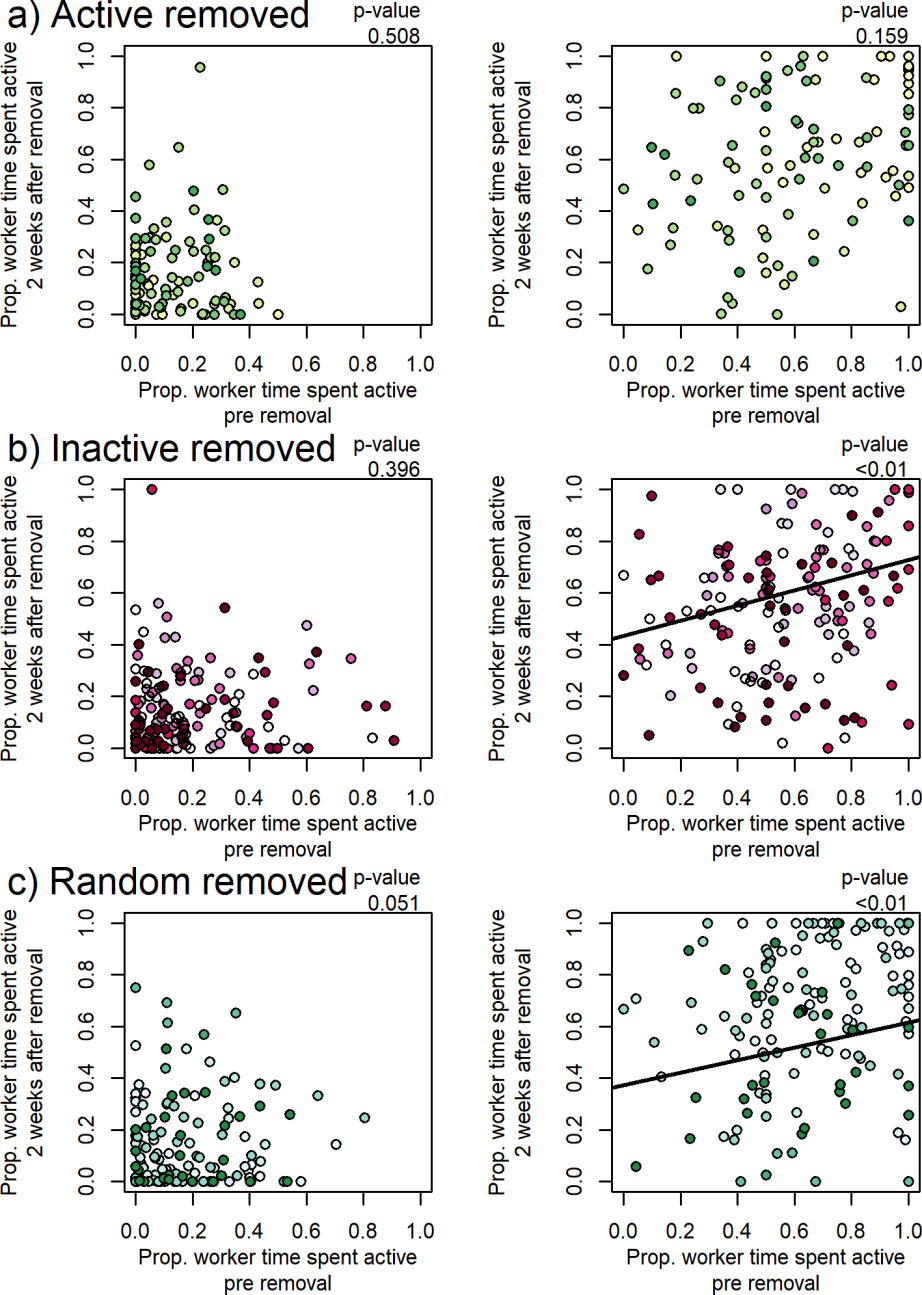
a) Time spent inactive pre-removal predicts time spent inactive post-removal when ‘inactive’ and randomly selected workers are removed (b and c), but not when ‘active’ workers are removed (a). Time spent active pre-removal did not predict time spent active post-removal for any treatment (a, b and c). Colored points highlight different colonies. Marginal (variance explained by fixed effects, i.e. by time spent active/inactive pre and post removal) and Conditional (total variance explained by fixed effects and random effects, i.e. including between-colony differences) R^2^: a) Active Removal—Activity: Marginal R^2^ = 0.007, Conditional R^2^ = 0.120; Inactivity: Marginal R^2^ = 0.024, Conditional R^2^ = 0.231; b) Inactive Removal—Activity: Marginal R^2^ = 0.005, Conditional R^2^ = 0.130; Inactivity: Marginal R^2^ = 0.081, Conditional R^2^ = 0.337; c) Random Removal—Activity: Marginal R^2^ = 0.018, Conditional R^2^ = 0.658; Inactivity: Marginal R^2^ = 0.048, Conditional R^2^ = 0.495. *Model: LMM, fixed: Worker activity/inactivity 2 weeks post ~ Worker activity/inactivity pre, random: Colony.

For individual workers, pre-removal activity levels did not predict post-removal (2 weeks) activity levels for any removal treatment. However, pre-removal inactivity levels were positively correlated to post-removal inactivity levels when ‘inactive’ and random workers were removed (Fig 4). This indicates that worker inactivity may be more consistent than worker activity or worker time spent wandering (perhaps looking for tasks), which is consistent with previous work [50]. This could result from the inclusion of tasks with low repeatability such as grooming and trophallaxis in the ‘active’ tasks [58].

**Fig 5:**
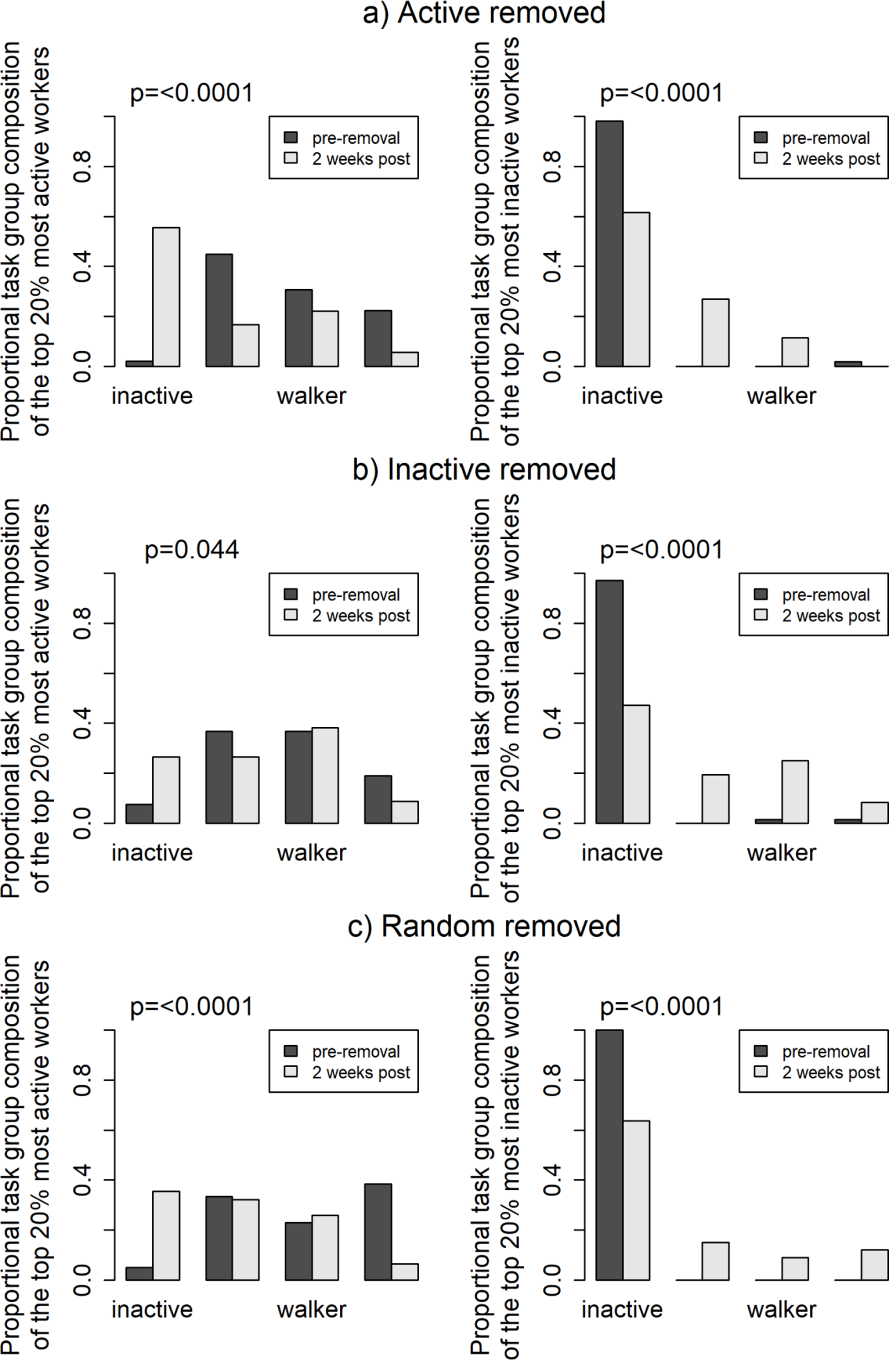
a) (left) Prior to ‘active’ worker removal, the 20% most active workers were mainly from the Nurse, Walker, and Forager task groups, but 2 weeks after removals the most active workers were mainly from the inactive and Walker task groups. b) (right) Prior to ‘inactive’ worker removal, the 20% most inactive workers were solely from the inactive task group, while 2 weeks after removals the most inactive workers were still mainly from the inactive task group, but there were also Nurses and Walkers (Fisher’s exact test, p<0.0001). c) After removal of random workers, colonies had (left) more inactive workers and less foragers in the top 20% most active, and (right) less inactive workers and more of each other task group in the 20% most inactive workers. * Fisher’s exact tests comparing frequencies of task groups pre- and 2 weeks post-removal.

The 20% most active workers before ‘active’ worker removal consisted mainly of workers from the Nurse and Forager task groups, while after removals the most active workers were mainly composed of workers from the Inactive and Walker task groups (remember that task groups reflect the role of workers pre-removal; Fig 5A). While there were Inactive workers in the top 20% active workers post-removal in all treatments, the presence of Inactives in the 20% most active was much greater in the active worker removals (Fig 5A—left) than in random or inactive removals (Fig 5B and 5C—left). Furthermore, the 20% most active workers after removal of random workers were significantly less active than pre-removal (Fig 2C—left). Therefore, the Inactive workers present in the 20% most active workers after random worker removal were overall less active than the Inactive workers that make up the 20% most active workers after active worker removal.

In the ‘inactive’ worker treatment, the 20% most inactive workers (top 20^th^ percentile) pre-removal were solely from the Inactive task group (as determined by hierarchical clustering analysis). Post-removal, the most inactive workers were still mainly from the Inactive task group, but also included some Nurses, Walkers and Foragers (Fig 5B). There was a small increase in Nurses, Walkers and Foragers in the 20% most inactive workers post-removal in all removal treatments (Fig 5 - right). Mean worker inactivity of the remaining 20% most inactive workers post inactive removal was significantly lower than that of the removed inactive workers (Fig 2C—right). This suggests that, although the 20% most inactive post-removal included Nurses, Walkers, and Foragers, these likely did not decrease their activity level to compensate for removed Inactives.

## Discussion

In this study, we test whether colonies of *Temnothorax rugatulus* ants maintain certain proportions of highly active and highly inactive workers in the colony. We show that colonies maintained pre-removal activity levels after active workers were removed, despite calculations showing that activity should have been significantly reduced had the colony not compensated for the loss of active workers. We also show that it is the previously inactive workers who became active subsequent to the removal of ‘active’ workers. This constitutes evidence for the hypothesis that inactive workers function as a reserve against worker loss and is relevant to the general question of task organization and how the colony responds to changes in task demands, including by task switches and switches to being active [55,77]. Conversely, colony activity remained at higher levels, and inactivity decreased, after ‘inactive’ workers were removed. Thus, the colony did not appear to compensate for the removal of inactive workers. Mean worker activity and inactivity levels were not affected by the removal of random workers.

### Inactive workers act as ‘reserve’ labor

Overall colony activity level was reestablished within 1 week of removals, and ‘active’ workers were effectively replaced by workers that increased their activity to levels comparable to those of removed ‘active’ workers. Replacement occurred relatively rapidly (<1 week) and was maintained 2 weeks after the removal of ‘active’ workers. This suggests that there are mechanisms ensuring a certain proportion of active workers within the colony.

Our data also showed that the 20% most active workers pre-removal were mainly composed of workers from the Nurse and Forager task groups, while 2 weeks post-removal, the most active workers were mainly workers who had previously (pre-removal) been in the Inactive task and Walker task groups. This suggests that workers that were inactive pre-removal effectively replaced lost ‘active’ workers. Interestingly, it may be that Walkers also play a similar role. This supports the hypothesis that inactive workers (and possibly ants that are wandering around with no clear task) are ‘reserves’ that serve as an auxiliary labor force that can help the colony react quickly to workload increases. Although this is the most commonly cited explanation for inactivity, evidence supporting it had been lacking. In fact, each previous study that explicitly set out to test the reserve worker hypothesis by increasing workload [23,30,47] or removing active workers [61,62,64] failed to support it, instead showing that either colonies do not adjust to changes in workload, or workers other than inactives increase their activity to compensate for the increased workload.

The only study that has effectively increased inactive worker activity levels did so by removing all but the most inactive workers in colonies of the ant *Myrmica kotokui* [66]. This study proposes that inactive workers are workers that have high response thresholds that only become active when all lower threshold workers are removed. A recent modeling study also suggested that if such highly inactive, high-threshold workers exist, they may be part of an adaptive strategy in social insects to mitigate the effects of large scale disturbances where most of the workers in the colony may be lost [67], though it is unclear what these disturbances may be or how frequently they occur in natural populations.

Nonetheless, the ant studied here, *Temnothorax rugatulu*s, may be the first species discovered in which inactive workers directly respond to the loss of active workers by increasing their activity levels to levels comparable to those of the removed workers, effectively taking over as the most active workers within the colony. This indicates that (at least some) inactive workers are not incapable of working when needed. It also suggests that, in the ant *Temnothorax rugatulus*, inactive workers may be part of an adaptive strategy to rapidly allocate workers to novel tasks and changing workloads thereby contributing to colony flexibility and robustness.

### Removed inactive workers are not replaced

When the 20% most inactive workers were removed, mean worker inactivity levels and the inactivity levels of the most inactive workers remained lowered both 1 and 2 weeks after removals. This suggests that, contrary to ‘active’ workers, ‘inactive’ workers are not replaced if lost. Thus, the replacement of ‘active’ workers likely does not result from random fluctuations in activity level for all workers, in which case all removed workers would be replaced without the need for specific mechanisms. Both pre and post-removal of ‘inactive’ workers, the 20% most inactive workers are largely composed of workers from the Inactive task group, suggesting that workers are not switching from other task groups to replace the removed ‘inactive’ workers.

### Removals minimally disrupt normal colony function

When randomly selected workers were removed, there were no significant effects on mean worker activity or mean worker inactivity. A slight trend of decreased activity and increased in inactivity occurred in the 1^st^ week, but after 2 weeks, both mean worker activity and inactivity appear to have regained levels comparable to pre-removal levels. Additionally, the 20% most active workers decreased their activity levels 1 week after removals and remained lower than pre-removal levels even after 2 weeks, though there is a slight, non-significant increase between 1 week and 2 weeks. Broadly, random worker removals have minimal effects on normal colony function as colonies maintain their activity levels post-removal, though there is disruption in the activity levels of the most active workers.

All removals seem to have cause an increase in Inactive workers among the 20% most active workers, though the effect is greatest when active workers were removed, followed by random removals, and then inactive removals. Removals also lead to decreases in Inactives in the 20% most inactive post-removal. The proportion of Inactives in the 20% most inactive was slightly smaller in the inactive removal treatment than in the active or random removals which should be expected when removed inactive workers are not replaced. Thus, the disturbance produced by worker removals seems to cause a low-level reallocation of worker activity levels, but the overall effect of the treatments (Inactives effectively replacing lost active workers, and the lack of replacement of lost inactives) can still be detected.

### ‘Reserve’ workers and colony flexibility

Group flexibility allows collective units to quickly adjust to changing environments. Colony-level flexibility is broadly thought to be attained either through highly flexible workers (i.e., generalists) that can switch between tasks when needed (but have high cognitive and sensory costs) or by highly specialized workers (inflexible by definition) allocated to various tasks as needed (high efficiency offset by cost of learning or morphological adaptations, e.g., large mandibles in soldiers). Here we show that colony flexibility can be attained by task-less workers that effectively function as ‘task-buffers’.

Although our results suggest that there are mechanisms in place for maintaining some colony homeostasis in activity level, we do not know what these mechanisms are. Our results are consistent with task allocation mechanisms that allow for flexible reallocation of workers to changing task demands such as response thresholds [18,78], foraging for work [79], and interaction rate mediated task allocation [80]. Mechanisms such as age/temporal polyethism [81,82] and task allocation based on body size (alloethism) [83] which are functionally inflexible (reversions are possible in temporal polyethism but require longer timescales) are less likely to be at play.

Reserve workers (i.e. task-less workers that serve as pool of spare workers) have only been tested for in a handful of species (honey bees, bumble bees, Polistes wasps [23,30,47,61,62]) that may rely more strongly on less flexible task allocation strategies, and this may have been the reason that inactive workers in these species did not appear to quickly pick up needed tasks. As such, we still do not have a sense of how prevalent ‘reserves’ may be across social insects. We can predict that colonies having evolved in unpredictable and high risk environments may have more selective pressure to maintain a pool of workers to compensate for worker loss. Additionally, we know that larger groups tend to have less inactive workers [84] which may indicate that larger groups have proportionally less need for inactive ‘reserves’. Of course, at this point we can only speculate and much additional research is needed to gain a broader understanding of ‘reserves’ as part of a task allocation strategy.

### ‘Reserves’ in other complex systems

The problem of adjusting supply to demand is a common one and the strategy of maintaining a reserve to deal with this is not unique to social insects [85]. For example, supply chains maintain stockpiles of products (warehousing or ‘slack’) to avoid shortages as demand increases [86–88], employers often employ contingent workers from external labor supply agencies to deal with changing demand [88], and computer systems perform better when they allow for a reserve of processing power (buffering) [89,90]. However, the problem faced by all of these systems is how to optimally organize the supply or reserve workforce such as to minimize the costs of maintaining these reserves. This involves making predictions about the future state of the system (e.g., what future supply and demand will be) and costs associated with either over- or underestimating supply and demand.

There does not appear to be a consistently optimal proportion of resources allocated to reserves across systems (e.g. the proportion of flexible workers in countries (temporary, standby, replacement, and other such workers) can account for 6.6%-10.7% of their active labor force [88], while in social insects between 50–70% of workers are inactive at any time [84]). This is not surprising, because the optimal amount of ‘reserves’ will be highly dependent on the predictability and variability of the environment; i.e., stable and predictable systems will require less of a buffer than systems where demand fluctuates widely and unpredictably. Thus, human organizations may have less reserves because they face more predictable environments than social insects, or perhaps human organizations do not sufficiently account for variation and should be employing additional flexible workers to optimally deal with varying environments.

Social insects face a range of predictable environmental fluctuations (e.g., regular cycles of brood production [91,92]) as well as unpredictable ones (e.g., weather [93], food availability [94], predation [95], pathogens and parasites [96,97]). However, the main problem of ants is competition (exploitation, interference, and apparent) with other ants (generally [98–102] and specifically in the genus *Temnothorax* [103,104]). However, we actually know very little about how much workload (demand) fluctuates in natural in social insect colonies, nor how colonies adjust their available workforce (supply) to these changing demands. For example, for most social insects we know very little about how food or other resource availability fluctuates in natural conditions, or how commonly workers are lost to causes other than old age, such as predation or natural events. We also know very little about the costs of inaccurately predicting how either supply or demand will vary in the future.

### Conclusion

Here we show that that colonies of *Temnothorax rugatulus* ants possess mechanisms ensuring the presence, and if necessary relatively quick replacement, of highly active workers, but not of inactive workers. Although inactive worker may serve a function within the colony, such as acting as food stores [58], or as a reserve labor force, our results suggest that these may not be continuously necessary tasks that require mechanisms to quickly reestablish their occurrence when workers are lost. On the other hand, if nurses and foragers (which are the main components of the ‘actives’) are lost, the colony seems to have mechanisms in place to quickly replace them and ensure that these tasks are kept up at comparable levels. This makes sense because without foraging and brood care, workers and brood won’t be fed, which will likely incur a large fitness cost to the colony very quickly. Thus, it seems that colonies do not seek to maintain homeostasis of colony activity in general, but rather that worker replacement depends on the immediate necessity of the task.

Although inactives may serve a function for the colony, there appear to be no mechanisms ensuring that a set proportion of the colony be dedicated to this task. Instead, workers allocated to ‘reserves’ may be determined by worker age and physiology corresponding to described traits of inactive workers (i.e., young reproductives or young ‘repletes’ who may not be well suited to work [58]). Thus inactives likely result from other age-related processes such as inexperience, physical vulnerability, degraded physiologies, decreased metabolic rates and immune function, etc. [58], or worker turnover frequency being decoupled from the frequency of fluctuations in colony workload [17]. In these cases, the mechanisms creating inactive workers are acting on much longer timescales and so removed inactive workers may only be replaced when the colony produces more workers.

We also showed for the first time that inactive workers can act as a ‘reserve’ labor force, effectively replacing lost active workers. Although this hypothesis is often proposed to explain highly inactive workers in social insect colonies, previous studies that have sought to test it have rejected it. This discrepancy suggests that inactivity likely has different causes and functions in different species of social insects.

## Supporting information

S1 Table List of colonies used in this study, removal treatments applied, and relevant demographic data. (DOCX)

S2 Table Mean activity/inactivity levels of colonies (colony-level activity = per colony mean of mean worker time spent on tasks) and removed workers (mean of mean worker time spent on tasks for removed workers) for each removal treatment. (DOCX)

S1 Fig (top) Distribution of mean activity and inactivity levels across colonies (mean of worker activity and inactivity levels for each colony as a data point) and (bottom) distribution of mean activity and inactivity levels across workers (mean of observed activity and inactivity levels for each worker as a data point; excludes workers only observed once).
(DOCX)

S2 Fig Removal of ‘active’ workers and ‘inactive’ significantly decreased mean worker activity and inactivity levels respectively (upper left and upper right), while removal of random workers should not significantly affect mean worker activity or inactivity (bottom left and right). Figures show comparisons of activity and inactivity levels of whole colonies (i.e. mean activity levels of all workers in a colony) pre-removal to the calculated mean colony activity level of colonies without the workers that would later be removed (i.e. mean activity levels of all workers in a colony minus the removed workers). Because of lost stored samples, not all removed workers were identified, therefore these analyses only include a subset of all data. *Wilcoxon Signed-Rank Test: Colony activity/inactivity level with removed workers vs. colony activity/inactivity level without removed workers.
(DOCX)
