# Supplementary material for: Who needs ‘lazy’ workers? Inactive workers act as a ‘reserve’ labor force replacing active workers, but inactive workers are not replaced when they are removed
Source: PLoS One. 2017 Sep 6;12(9):e0184074. doi: 10.1371/journal.pone.0184074 (PMC5587300; doi:10.1371/journal.pone.0184074)
Supplement: S1 Table — (DOCX) [file pone.0184074.s001.docx]

S1 Tab: List of colonies used in this study, removal treatments applied, and relevant demographic data.

| Colony | Removal Treatment | Collection date | Number of ants observed | | | |
| --- | --- | --- | --- | --- | --- | --- |
|  |  |  | Min | Max | Mean | SD |
| Jul14_11 | Active | Jul-14 | 85 | 105 | 97.0 | 10.58 |
| Jul14_14 | Active | Jul-14 | 33 | 34 | 33.7 | 0.58 |
| Jul14_16 | Active | Jul-14 | 76 | 81 | 78.3 | 2.52 |
| Jul14_19 | Active | Jul-14 | 45 | 47 | 46.0 | 1.00 |
| Jun14_7 | Active | Jun-14 | 53 | 57 | 54.7 | 2.08 |
| Jul14_18 | Inactive | Jul-14 | 52 | 55 | 53.3 | 1.53 |
| Jul14_20 | Inactive | Jul-14 | 51 | 52 | 51.7 | 0.58 |
| Jul14_21 | Inactive | Jul-14 | 23 | 31 | 27.0 | 4.00 |
| Jul15_1 | Inactive | Jul-15 | 65 | 66 | 65.3 | 0.58 |
| Jul15_2 | Inactive | Jul-15 | 25 | 25 | 25.0 | 0.00 |
| Jul15_3 | Inactive | Jul-15 | 43 | 44 | 43.3 | 0.58 |
| Jul15_4 | Inactive | Jul-15 | 31 | 33 | 31.7 | 1.15 |
| Jul15_5 | Inactive | Jul-15 | 81 | 88 | 85.0 | 3.61 |
| Jun14_6 | Inactive | Jun-14 | 71 | 97 | 88.0 | 14.73 |
| Jul14_12 | Random | Jul-14 | 92 | 94 | 92.7 | 1.15 |
| Jul14_13 | Random | Jul-14 | 71 | 76 | 74.3 | 2.89 |
| Jul14_17 | Random | Jul-14 | 112 | 116 | 114.3 | 2.08 |
| Jun14_1 | Random | Jun-14 | 15 | 16 | 15.7 | 0.58 |
| Jun14_4 | Random | Jun-14 | 48 | 52 | 50.3 | 2.08 |
| Jun14_5 | Random | Jun-14 | 137 | 138 | 137.7 | 0.58 |
