# Supplementary material for: Who needs ‘lazy’ workers? Inactive workers act as a ‘reserve’ labor force replacing active workers, but inactive workers are not replaced when they are removed
Source: PLoS One. 2017 Sep 6;12(9):e0184074. doi: 10.1371/journal.pone.0184074 (PMC5587300; doi:10.1371/journal.pone.0184074)
Supplement: S2 Table — (DOCX) [file pone.0184074.s002.docx]

S2 Tab: Mean activity/inactivity levels of colonies (colony-level activity = per colony mean of mean worker time spent on tasks) and removed workers (mean of mean worker time spent on tasks for removed workers) for each removal treatment.

| **Removal** | Activity | | | | Inactivity | | | |
| --- | --- | --- | --- | --- | --- | --- | --- | --- |
|  | Removed | | Colony | | Removed | | Colony | |
|  | **Mean** | s.d. | **Mean** | s.d. | **Mean** | s.d. | **Mean** | s.d. |
| Active | **0.42** | 0.16 | **0.18** | 0.07 | **0.28** | 0.17 | **0.55** | 0.03 |
| Inactive | **0.06** | 0.08 | **0.16** | 0.09 | **0.75** | 0.28 | **0.64** | 0.18 |
| Random | **0.20** | 0.00 | **0.17** | 0.05 | **0.57** | 0.10 | **0.63** | 0.17 |
