# Supplementary material for: Who needs ‘lazy’ workers? Inactive workers act as a ‘reserve’ labor force replacing active workers, but inactive workers are not replaced when they are removed
Source: PLoS One. 2017 Sep 6;12(9):e0184074. doi: 10.1371/journal.pone.0184074 (PMC5587300; doi:10.1371/journal.pone.0184074)
Supplement: S1 Fig — (top) Distribution of mean activity and inactivity levels across colonies (mean of worker activity and inactivity levels for each colony as a data point) and (bottom) distribution of mean activity and inactivity levels across workers (mean of observed activity and inactivity levels for each worker as a data point; excludes workers only observed once). (DOCX) [file pone.0184074.s003.docx]

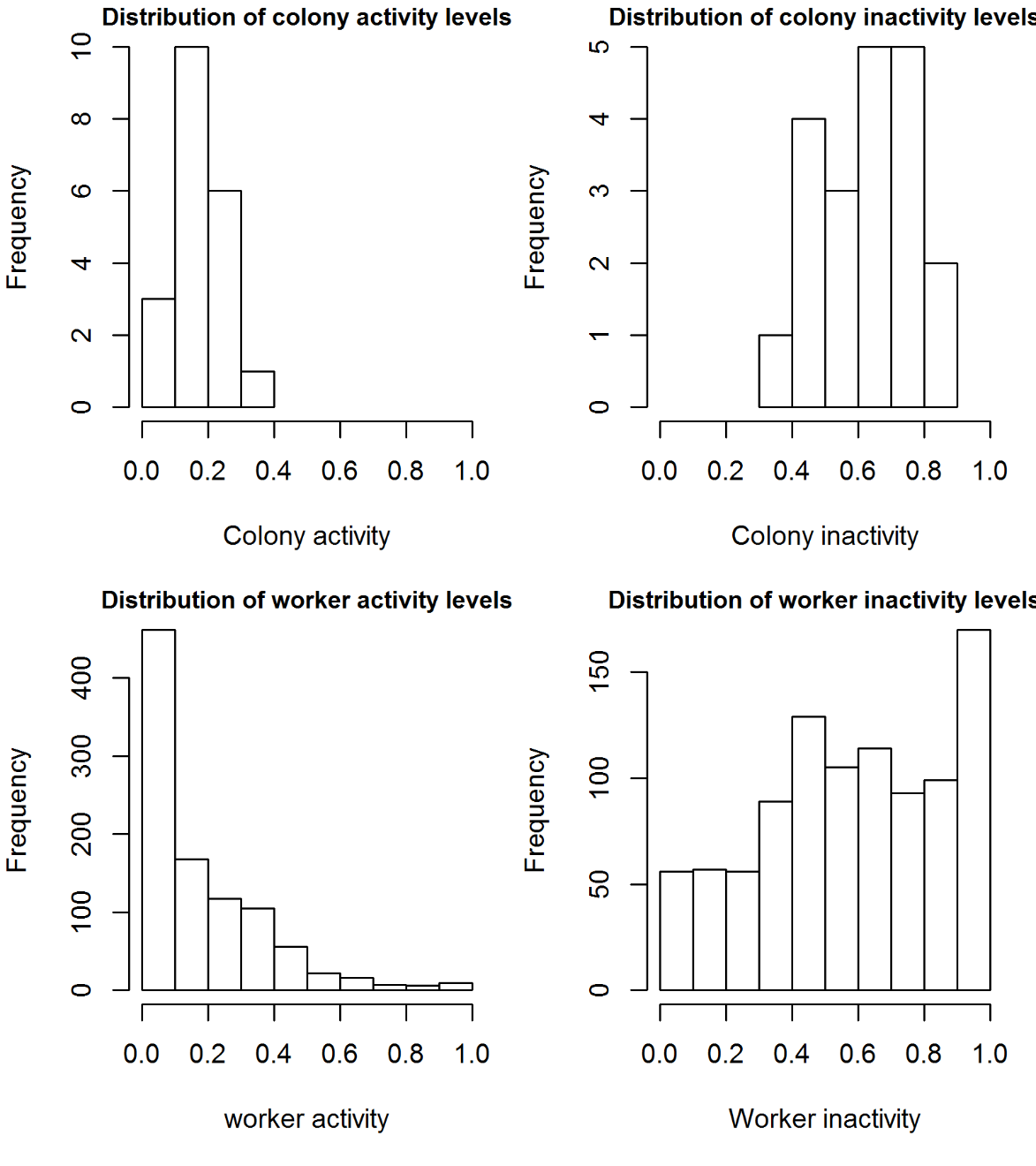


S1 Fig: (top) Distribution of mean activity and inactivity levels across colonies (mean of worker activity and inactivity levels for each colony as a data point) and (bottom) distribution of mean activity and inactivity levels across workers (mean of observed activity and inactivity levels for each worker as a data point; excludes workers only observed once).
