# Supplementary material for: Who needs ‘lazy’ workers? Inactive workers act as a ‘reserve’ labor force replacing active workers, but inactive workers are not replaced when they are removed
Source: PLoS One. 2017 Sep 6;12(9):e0184074. doi: 10.1371/journal.pone.0184074 (PMC5587300; doi:10.1371/journal.pone.0184074)
Supplement: S2 Fig — Figures show comparisons of activity and inactivity levels of whole colonies (i.e. mean activity levels of all workers in a colony) pre-removal to the calculated mean colony activity level of colonies without the workers that would later be removed (i.e. mean activity levels of all workers in a colony minus the removed workers). Because of lost stored samples, not all removed workers were identified, therefore these analyses only include a subset of all data. *Wilcoxon Signed-Rank Test: Colony activity/inactivity level with removed workers vs. colony activity/inactivity level without removed workers. (DOCX) [file pone.0184074.s004.docx]

a) Active removed

c) Random removed

b) Inactive removed


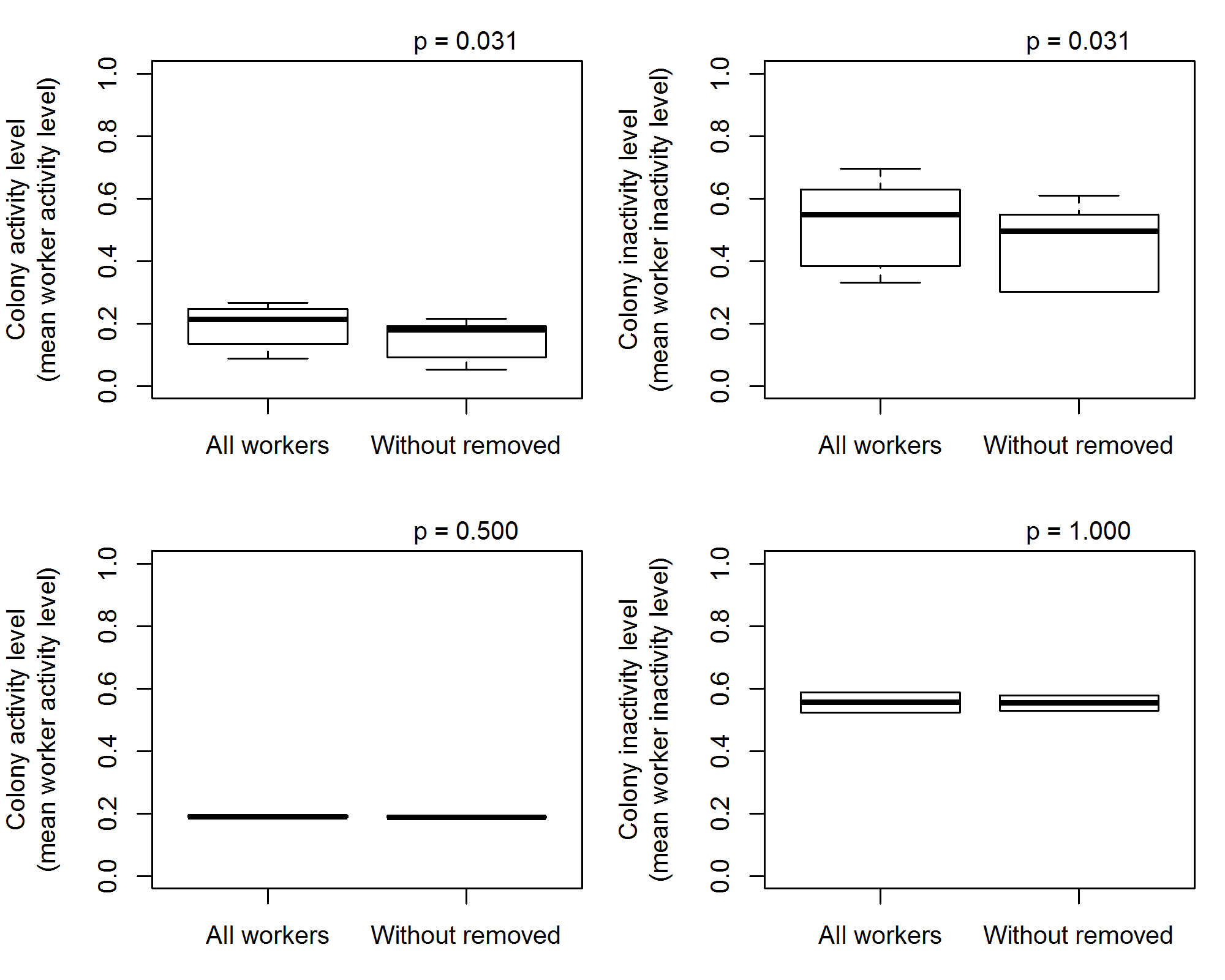


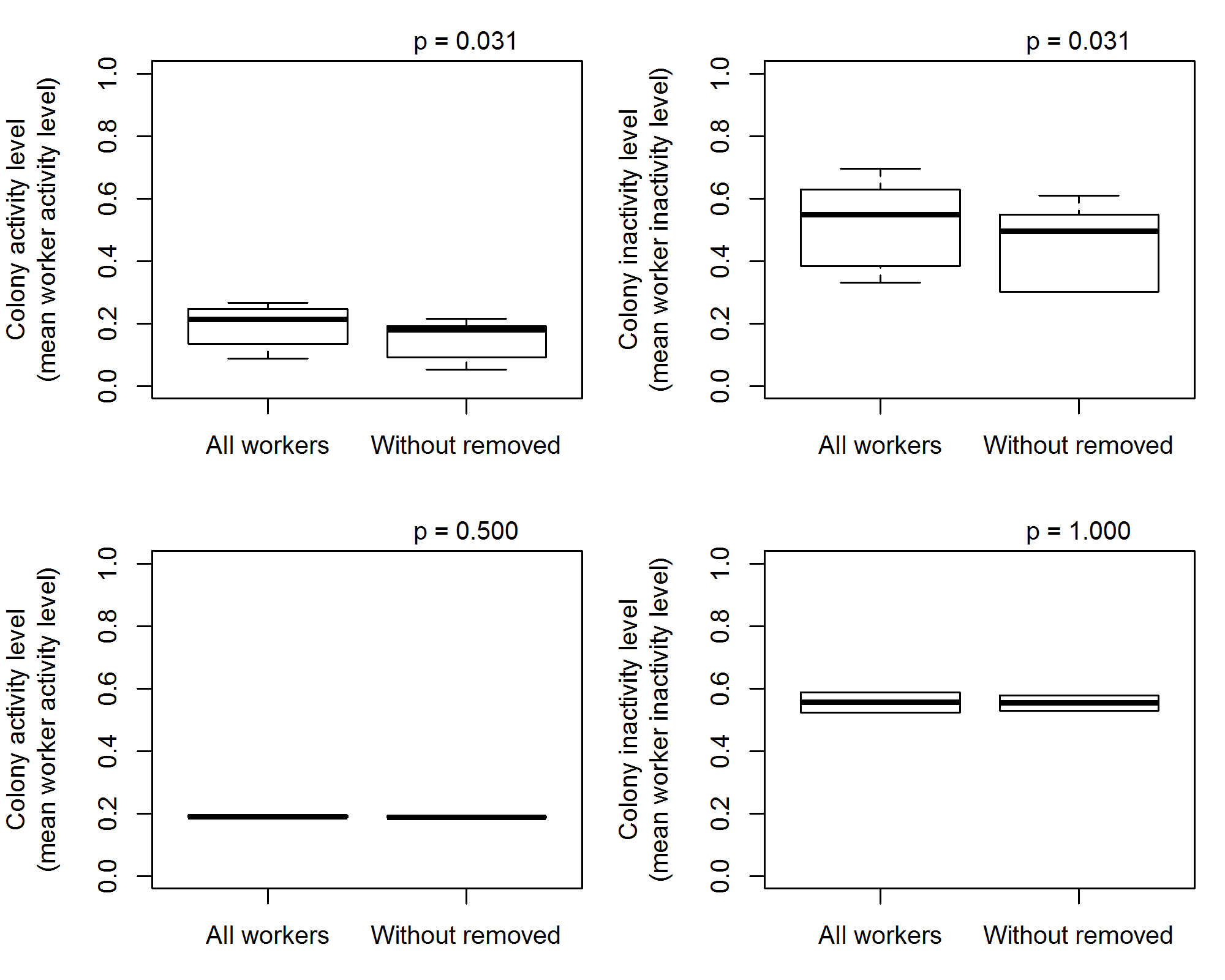


S2 Fig: Removal of ‘active’ workers and ‘inactive’ significantly decreased mean worker activity and inactivity levels respectively (upper left and upper right), while removal of random workers should not significantly affect mean worker activity or inactivity (bottom left and right). Figures show comparisons of activity and inactivity levels of whole colonies (i.e. mean activity levels of all workers in a colony) pre-removal to the calculated mean colony activity level of colonies without the workers that would later be removed (i.e. mean activity levels of all workers in a colony minus the removed workers). Because of lost stored samples, not all removed workers were identified, therefore these analyses only include a subset of all data.

*Wilcoxon Signed-Rank Test: Colony activity/inactivity level with removed workers vs. colony activity/inactivity level without removed workers
